# Delays in Diagnosis and Treatment in Patients Underwent Endobronchial Ultrasound-Transbronchial Needle Aspiration (EBUS-TBNA)

**DOI:** 10.1155/2022/7546012

**Published:** 2022-07-18

**Authors:** Emine Gülçek, Murat Yalçınsoy, İlham Gülçek, Arzu Nakış Güven, Hilal Ermiş, Zeynep Ayfer Aytemur

**Affiliations:** ^1^Department of Pulmonary Medicine, Inonu University Medical Faculty, Turgut Ozal Medical Center, Malatya, Turkey; ^2^Department of Thoracic Surgery, Inonu University Medical Faculty, Turgut Ozal Medical Center, Malatya, Turkey

## Abstract

**Objectives:**

Endobronchial ultrasound-guided transbronchial needle aspiration (EBUS-TBNA) has been recognized as the first method of choice in the diagnosis of mediastinal and hilar lesions. Although the procedure is commonly used, there is no study assessing its contribution to the duration required for diagnosis and treatment. In this study, we aimed to determine the extent of diagnosis and treatment delays when using the EBUS-TBNA procedure and to address the possible factors contributing to these delays.

**Materials and Methods:**

The demographic data, pathological diagnosis, need for additional procedures, symptoms, presenting complaints, and the time until the beginning of treatment were recorded retrospectively in all patients who had undergone EBUS-TBNA.

**Results:**

A total of 134 patients (mean age 60.7 ± 12 years, M/F: 78/56) were included. Delay of the patients was found in 60.4% (*n* = 81), delayed referral in 35.8% (*n* = 48), diagnosis delays in 84.3% (*n* = 113), treatment delays in 38.8% (*n* = 52), and total delay in 73.1% (*n* = 98) of the patients. A statistically significant association was found between referral delay and total delay with age groups (*p*=0.006) and between patient delay and the presence of symptoms (*p*=0.027). EBUS-TBNA was found to have the lowest effect among all delay parameters (*β*: 0.104, *p* < 0.001) in the regression analysis. When diagnosis times' subgroups were compared, EBUS-TBNA was found to have the least effect (correlation coefficient: 0.134, *p*=0.004).

**Conclusion:**

We found that approximately ¾ of the patients had a delay and this is not acceptable in real terms. Considering that the patient burden is increasing day by day, it is necessary to make a radical change in health care or a change in strategy in order to prevent delays. EBUS-TBNA, which is in the diagnosis delay subgroup, is less invasive and accelerates the process.

## 1. Introduction

A failure in the nature of the long diagnostic process in mediastinal diseases which employs a complex algorithm may cause delays in diagnosis and treatment, consequently resulting in the worsening of the disease course or eliminating the patient's chance to receive a cure. Preventing delay in diagnosis and treatment is very important in terms of the treatment of the patient and both health workload and cost. Diagnosis and treatment delays may stem from the capability of the center or from the patients' noncompliance. Depending on the skill and expertise of the performing endoscopist, both the diagnostic value and the complication rate of the procedure vary [1]. Diagnosis and treatment delays, which are common in all diseases, may be caused by the patient or the clinicians. However, the contribution of EBUS-TBNA to delays in the diagnosis and treatment is currently unknown. Therefore, we aimed to investigate the role of EBUS-TBNA procedure, which has been preferred as the first method of choice in recent years, in diagnosis and treatment delays.

## 2. Materials and Methods

### 2.1. Patients

Patients that underwent EBUS between March 2017 and December 2019 at the chest diseases clinic of Inonu University Turgut Ozal Medical Center were included in our study retrospectively. Patients with missing data in their files and those could not be operated on due to complications were excluded from the study. Ethical approval was obtained from the Scientific Research and Publication Ethics Committee of Inonu University for the study (approval date/number: 2020/1126). Before the procedure, in accordance with the principles of our clinic, an informed consent form is obtained from each patient. In our center, EBUS is performed once a week under general anesthesia.

### 2.2. Definitions

The date when the patient's complaints first started, the first admission date to the doctor, the date of referral, the date of EBUS, the completion date of the pathological diagnosis, and the date of the start of the treatment were recorded from the patient files. Based on these dates, parameters regarding durations and delays were calculated.

#### 2.2.1. Definitions of the Time Periods

Admission time: the time between the beginning of the first complaint of the patient and the first admission to the doctor [2, 3].

Time for referral: the time between the first admission to a doctor and referral to our center.

Initial evaluation time: the time between the patient's initial evaluation and planning the EBUS procedure. Unlike other studies in the literature on diagnosis and treatment delays, in this study, the time for the initial evaluation period includes differential diagnostic laboratory examinations, radiological imaging, or interventional procedures performed by the pulmonologists in our center for the patients who are either consulted directly or by referral to our center or consulted from different clinics in the center, until the scheduling of the EBUS-TBNA date.

Time until EBUS-TBNA: the time between scheduling and performing the EBUS procedure. In our center, EBUS-TBNA is performed once a week under anesthesia, and the preoperative preparation period is included within the time until EBUS-TBNA.

Time until pathological diagnosis: the time period between EBUS and the completion of the pathological diagnosis.

Duration of diagnosis: the period from the patient's first admission to us until the diagnosis is completed. It consists of three subfeatures: initial evaluation time, time until EBUS-TBNA, and time until pathological diagnosis.

Time until the treatment start: the time before starting treatment after diagnosis has been completed [4, 5].

Overall time: the time from the patient's first complaint until the start of the treatment.

#### 2.2.2. Definitions of the Delay

For determining the delay times, the categorizations were made in accordance with the previous international studies in the literature.

Patient delay: when the patient admission period exceeds 30 days [3].

Referral delay: when referral time exceeds 2 weeks.

Diagnosis delay: when the duration of the diagnosis period exceeds 2 weeks.

Delay in treatment start: when the period until treatment start exceeds 2 weeks.

Clinician's delay: when the time between the first admission to the doctor and the treatment start is more than 6 weeks.

Overall delay: when the time from the first complaint of the patients until the start of the treatment is longer than 72 days [6, 7].

**Figure 1:**
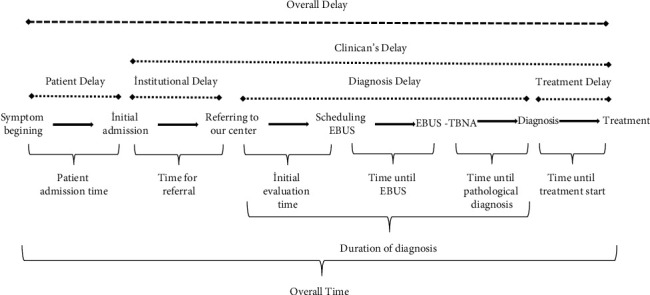
The schematic representation of the time periods and delay in the study.

The definition of time periods and delay subgroups is summarized in Figure 1.

### 2.3. Data Collection

Clinical data of the patients including demographic data (age, gender, and use of tobacco), comorbidities, symptoms, laboratory data, pathological data, and information about treatment were recorded. In order to calculate the durations and delays, the time of the symptom onset, the referral time (if applicable), the time for interventional or diagnostic procedures, the completion time of the pathological diagnosis of the sample, and the beginning of the treatment times were recorded.

### 2.4. Statistical Analysis

Data analysis was performed using IBM SPSS software v. 25.0 for Windows. For normality analysis, the Shapiro–Wilk test, histogram distribution, and skewness-kurtosis parameters were used. Descriptive statistics are shown as mean ± standard deviation for variables with normal distribution, as median (Min–Max) for variables with nonnormal distribution, and as number of cases and percentage for nominal variables. The chi-square test and Fisher's exact test were used to analyze the relationship between categorical variables. In order to determine the relationship between continuous variables, the Spearman correlation test was used when the variables were nonparametric. Multivariate linear regression analysis was used to calculate the strength of the effect of more than one independent variable on a dependent variable. A *p* value of less than 0.05 (*p* < 0.05) was considered statistically significant.

## 3. Results

### 3.1. Patient Characteristics

We assessed 134 patients that underwent EBUS. The characteristics of these patients are summarized in Table 1.

The calculated time periods of the patients and the extent of delays are given in Table 2. The highest delays were observed during the diagnosis process.

### 3.2. Patient Delay

Patient delay was present in 81 (60.4%) patients. No significant difference was found between gender, age groups, presence of comorbidity, diagnosis, smoking, and patient delay. There was a significantly higher patient delay in asymptomatic patients when compared to symptomatic patients (*p*: 0.027) (Table 3). The subgroup analyses revealed that EBUS-TBNA performed in asymptomatic patients resulted in malignant diagnosis for 36% (*n*: 9) of the patients, 48% (*n*: 12) as benign diagnosis, and 16% (*n*: 4) as nondiagnostic.

### 3.3. Referral Delay

The referral delay was present in 48 (35.8%) patients, whereas 64.2% (*n*: 86) of the patients were referred to our center in less than two weeks. No significant difference was found between gender, presence of comorbidity, smoking status, presence of symptoms, and delayed diagnosis and referral. Delay in referral was 20% (*n*: 4) in patients under 50 years old, 21% (*n*: 6) in patients aged 50–60 years, 53% (*n*: 29) in patients aged 60–70, and 30% (*n*: 29) in patients aged more than 70 years. A significant relationship was shown between referral delay and the age groups (*p* : 0.006) (Table 3).

### 3.4. Diagnosis Delay

The delay in diagnosis was found as 84.3% (*n*: 113). Among the diagnostic period subgroups, the longest time was the period until the pathological results. No significant difference was found between gender, age groups, presence of comorbidity, smoking, presence of symptoms, additional procedures, the diagnosis of the patient, and the diagnosis delay.

### 3.5. Delay in Treatment Start

The presence of delay until the treatment start was 38.8% (*n*: 52). No significant difference was found between gender, age groups, presence of comorbidity, smoking, presence of symptoms, additional procedures, diagnosis of the patient, and the diagnosis delay.

### 3.6. Overall Delay

The overall delay of patients from the onset of symptoms to the start of the treatment was 73.1% (*n*: 98). No difference was found between sex, presence of comorbidity, smoking, presence of symptoms, additional procedures, diagnosis, and delay in diagnosis; however, a significant relationship was observed for age groups and overall delay (*p* : 0.042).

In order to determine the effect of each parameter of the delay times on the overall delay, the multivariate linear regression analysis with the overall delay selected as a dependent variable yielded in standardized beta coefficients as 0.593 for patient admission time, 0.415 for referral time, 0.262 for initial evaluation time, 0.104 for time until EBUS-TBNA, 0.269 for time until pathological diagnosis, and 0.424 for time until treatment start, respectively. The effect of each parameter on the overall delay was found to be statistically significant (*p* < 0.001) (Table 4).

The correlation analysis of the subfeatures that constitute the time for diagnosis with the duration of diagnosis period resulted in the correlation coefficients as follows: the correlation coefficient for the initial evaluation time was 0.599, for the time until EBUS-TBNA was 0.134, and for the time until pathological diagnosis was 0.611 (Table 5).

## 4. Discussion

Delays in diagnosis and treatment constitute an important health problem for all diseases. It is critically important for malignant patients; delayed treatment of benign diseases can cause serious problems. To our knowledge, this study is the first study assessing the amount of delays in patients who underwent EBUS. In our study, we found serious delays in all time parameters, especially in the diagnosis period. Considering the parameters affecting the diagnosis time, the time until EBUS-TBNA was the shortest, whereas the time until pathological diagnosis and the initial evaluation took the longest.

In a study by Koyi et al., the patient admission time was reported as 43 days [5]. In a systematic review examining the diagnosis and treatment delays in lung cancer, admission time was found to be 14 days [8]. In another study, the mean patient admission time was reported as 41 days [2], which is similar compared to our results. Another study with a similar patient admission time to our study was conducted by Ozlü et al. in our country where the median patient admission time was found as 30 days [9]. In the study of Forrest et al., the referral delay was found to be 29.6%, which was found to be associated with advanced disease and socioeconomic status [10]. In our study, patients aging between 60 and 70 had longer referral delays than other age groups. Again, for the same age group, the overall delay was also long and when evaluated together with the age group of 70, there was no significant difference. We suggest that the observed high rate of patient delay, referral delay, and overall delay in these age groups are related to the symptom status of the patients. 64% of asymptomatic patients were over 60 years old. Due to the absence of symptoms in these patient groups, there might be delays in admission to the health services, slow processing of the examinations, and prolonged referral times. The most important reason for patient delay is either the absence of symptoms or neglect. Delay in diseases that cause mediastinal hilar lymphadenopathy is a cause of serious morbidity and mortality [11, 12]. A high proportion of our asymptomatic patients, up to 36%, were diagnosed as malignant, and the delays that may occur in these patients are crucial as a factor increasing. For asymptomatic patients with mediastinal and hilar lymphadenopathy that will be referred for examination, it is important to warn the patient in order not to delay their admission to the referred health institution.

In a meta-analysis of 24 different studies with tuberculosis patients, the mean diagnosis time was found to be 69.3 days [13]. In a study of 1330 patients with lung cancer by Fernandez et al., the mean diagnosis time was found to be 19.8 ± 13.9 days [14]. In a recent survey analysis, the median time to diagnosis in cancer patients was found to be 11.05 days [15]. In our study, the diagnosis time and delay were found to be quite high compared to the literature, especially in malignant patients. When the subgroups of the duration of diagnosis were examined, the time until EBUS-TBNA was the shortest. Since there are no similar studies in the literature to ours, there is no similar diagnosis time categorization, but close classifications were used such as initial doctor delay and secondary doctor delay. In a study similar to this study, the second doctor's delay was used as a similar term to the initial evaluation period which was found to be 33 days on average and the median of 9 days. These are remarkably longer compared to the initial evaluation time we report in our study [5]. The initial evaluation and pathological evaluation seen take the longest time when considering the categorization in the diagnosis process. We think that the delay in the initial evaluation time is mainly due to the postponement of radiological examinations in our center. The pathological diagnosis delay is also due to the density of patients in our center. This is due to the fact that there is no referral chain in the health policy in our country and that direct admission can be made to the tertiary center. We think that the arrangements to be made in this direction will reduce the delays.

There are several studies about the duration until treatment in the literature. In the first studies on this subject by Finlay et al. [16] with 42 patients between 1992 and 1996, the median time until treatment was 31 days, and in a study by Liu et al. with 1394 patients, the median time until treatment was reported as 27 days [17]. Between 2006 and 2010, Forrest et al. reported the median value until the treatment period as 31 days in lung cancer patients [10]. Comparing these studies, it can be concluded that the time until the treatment is shorter using the EBUS-TBNA procedure. In another study with tuberculosis patients, the time until treatment start was reported as 7.9 days, which is shorter than ours [13]. Although we found a 38.8% delay in our study, it is seen that there is an acceptable delay when compared with the literature.

Our study has some limitations. First of all, our study has a retrospective design; therefore, sociodemographic data such as profession, educational status, and patient-related reasons that may cause delay could not be questioned. Second, the time obtained with EBUS-TBNA procedure was not compared with other methods such as mediastinoscopy. However, the current literature shows that using EBUS-TBNA provides results in a shorter time, making such a study design raising ethical issues. The final limitation is that our study was conducted within a single center; thus, some factors such as socioeconomic, cultural, and geographical differences that may affect delay could not be examined.

In conclusion, it is very important that this process is fast as well as provides the correct diagnosis and effective treatment of the patients in the provision of health services. In patients whose mediastinal and hilar lymphadenopathy etiology is investigated, being asymptomatic and being older cause a significant delay in the admission period. In our study, EBUS-TBNA covered the shortest duration of all periods. It will be possible to reduce delays at all stages with multidisciplinary work and more efforts of hospital management.

## Figures and Tables

**Table 1:** The characteristics of patients.

Variable	*n* (%)
Age, mean ± SD (years)	60.7 ± 12.1
Gender	
Female	56 (41.8)
Male	78 (58.2)
Smoking status	
Current smoker	39 (29)
Ex-smoker	38 (28)
Nonsmoker	57 (43)
Comorbidities	
Hypertension	46 (34.3)
Diabetes mellitus	22 (16.4)
COPD	21 (15.7)
CLD	21 (15.7)
Malign diseases	18 (13.4)
Chronic liver disease	4 (3.0)
CLF	3 (2.2)
Thyroid diseases	3 (2.2)
Rheumatic diseases	3 (2.2)
None	42 (31.3)
Charlson Comorbidity Index	
<3	40 (30)
3–6	62 (46)
>6	32 (24)
Lymph nodes by location, number	
2R	2 (0.9)
2L	1 (0.5)
4R	65 (8.30)
4L	22 (4.10)
7	64 (3.30)
10R	13 (2.6)
10L	1 (0.5)
11R	19 (9)
11L	11 (2.5)
12R	7 (3)
12L	6 (2)
Symptoms	
Cough	58 (43.3)
Shortness of breath	44 (32.8)
Chest pain	31 (23.1)
Sputum	29 (21.6)
Weight loss	20 (14.9)
Night sweats	17 (12.7)
Hemoptysis	12 (9.0)
Hoarseness	7 (5.2)
Weakness-fatigue	6 (4.5)
Difficulty swallowing	1 (0.7)
Asymptomatic	25 (18.7)
LN pathology	
Benign	47 (35.1)
Malignant	57 (42.5)
Nondiagnostic	30 (22.4)

**Table 2:** Durations of periods and amount of delays of the patients.

	Mean value ± standard deviation	Median value (Min–Max)	Delay presence (*n* (%))
Admission time (days)	40.9 ± 28.7	30 (0–150)	81 (60.4)
Time for referral (days)	19.1 ± 20.7	13.5 (1–120)	48 (35.8)
Time until the treatment start (days)	32.1 ± 17.9	29 (3–106)	113 (84.3)
Initial evaluation time	7.4 ± 12.6	0 (0–80)	
Time until EBUS-TBNA	5.0 ± 5.1	4 (0–26)	
Time until pathological diagnosis	19.8 ± 12.7	17 (3–80)	
Time until the treatment start (days)	15.5 ± 19.5	11 (0–110)	52 (38.8)
Overall time (days)	103.3 ± 49.4	95.5 (13–288)	98 (73.1)

**Table 3:** Risk factors associated with delay for five time periods in patients who underwent EBUS-TBNA.

Parameters	Patient delay		Referral delay		Diagnosis delay		Delay in treatment start		Overall delay	
	*n (%)*	*P*	*n (%)*	*p*	*n (%)*	*p*	*n (%)*	*p*	*n (%)*	*p*
Gender		0.681		0.478		0.914		0.648		0.229
Male	46 (59)		26 (33.3)		66 (84.6)		29 (37.2)		54 (69.2)	
Female	35 (62.5)		22 (39.3)		47 (83.9)		23 (41.1)		44 (78.6)	
Age		0.178		**0.006** ^*∗*^		0.394		0.078		**0.042** ^*∗*^
˂50 years	8 (40)		4 (20)		16 (80)		10 (50)		13 (65)	
50–60 years	17 (59)		6 (21)		25 (86)		11(38)		17 (59)	
60–70 years	35 (64)		29 (53)		44 (80)		15 (27)		41 (74)	
>70 years	21 (70)		9 (30)		28 (93)		16 (53)		27 (90)	
Charlson CI		0.462		0.151		0.795		0.161		0.371
˂3 points	21 (52.5)		10 (25)		34 (85)		16 (40)		26 (65)	
3–6 points	40 (64.5)		23 (37.1)		51 (82.3)		28 (45.2)		48 (77.4)	
6 points ˂	20 (67.5)		15 (46.9)		28 (87.5)		8 (25)		24 (75)	
Smoking		0.301		0.844		0.846		0.794		0.624
Nonsmoker	35 (61.4)		22 (38.6)		48 (84.2)		24 (42.1)		44 (77.2)	
Ex-smoker	26 (68.4)		13 (34.2)		33 (86.8)		14 (36.8)		26 (68.4)	
Active smoker	20 (51.3)		13 (33.3)		32 (82.1)		14 (35.9)		28 (71.8)	
Symptom		**0.027** ^*∗*^		0.344		0.544		0.260		0.695
Asymptomatic	20 (80)		11 (44)		20 (80)		7 (28)		17 (68)	
Symptomatic	61 (56)		37 (33.9)		93 (85.3)		45 (41.3)		81 (74.3)	
Diagnosis		0.113		0.588		0.321		0.807		0.617
Malignant	31(54.4)		20 (35.1)		45 (78.9)		21 (36.8)		41 (71.9	
Benign	27 (57.4)		15 (31.9)		42 (89.4)		20 (38)		33 (70.2)	
Insufficient diagnosis	23 (76.7)		13 (43.3)		26 (86.7)		11 (36.7)		24 (80)	
Additional procedures						0.981		0.062		0.439
None					81 (84.4)		42 (43.8)		72 (75)	
Present					32 (84.2)		10 (26.3)		26 (68.4)	

**Table 4:** Coefficients for the factors affecting the overall delay time using the linear regression model.

	Standardised beta coefficient	*p*
Admission time	0.593	<0.001
Time for referral	0.415	<0.001
Initial evaluation time	0.262	<0.001
Time until EBUS-TBNA	0.104	<0.001
Time until pathological diagnosis	0.269	<0.001
Time until the treatment start	0.424	<0.001

**Table 5:** The results of the correlation analysis of the duration of diagnosis with its subfeatures.

	Initial evaluation time	Time until EBUS-TBNA	Time until pathological diagnosis
Duration of diagnosis correlation coefficient	0.599	0.134	0.611
*P*	<0.001	0.004	<0.001

## Data Availability

All the data of our study are clearly available as both spss and excel files.
